# SmaRT2P: a software for generating and processing smart line recording trajectories for population two-photon calcium imaging

**DOI:** 10.1186/s40708-022-00166-4

**Published:** 2022-08-04

**Authors:** Monica Moroni, Marco Brondi, Tommaso Fellin, Stefano Panzeri

**Affiliations:** 1 Neural Computation Laboratory, Center for Neuroscience and Cognitive Systems, UniTn, Istituto Italiano Di Tecnologia, 38068 Rovereto, Italy; 2 Optical Approaches to Brain Function Laboratory, Istituto Italiano Di Tecnologia, 16163 Genoa, Italy; 3 Department of Biomedical Sciences-UNIPD, Università Degli Studi Di Padova, 35121 Padua, Italy; 4 Padova Neuroscience Center (PNC), Università Degli Studi Di Padova, 35129 Padua, Italy; 5 Department of Excellence for Neural Information Processing, Center for Molecular Neurobiology (ZMNH), University Medical Center Hamburg-Eppendorf (UKE), 20251 Hamburg, Germany

**Keywords:** Two-photon microscopy, Line scanning, Calcium imaging, Open-source software, Neuroinformatics

## Abstract

Two-photon fluorescence calcium imaging allows recording the activity of large neural populations with subcellular spatial resolution, but it is typically characterized by low signal-to-noise ratio (SNR) and poor accuracy in detecting single or few action potentials when large number of neurons are imaged. We recently showed that implementing a smart line scanning approach using trajectories that optimally sample the regions of interest increases both the SNR fluorescence signals and the accuracy of single spike detection in population imaging in vivo. However, smart line scanning requires highly specialised software to design recording trajectories, interface with acquisition hardware, and efficiently process acquired data. Furthermore, smart line scanning needs optimized strategies to cope with movement artefacts and neuropil contamination. Here, we develop and validate SmaRT2P, an open-source, user-friendly and easy-to-interface Matlab-based software environment to perform optimized smart line scanning in two-photon calcium imaging experiments. SmaRT2P is designed to interface with popular acquisition software (e.g., ScanImage) and implements novel strategies to detect motion artefacts, estimate neuropil contamination, and minimize their impact on functional signals extracted from neuronal population imaging. SmaRT2P is structured in a modular way to allow flexibility in the processing pipeline, requiring minimal user intervention in parameter setting. The use of SmaRT2P for smart line scanning has the potential to facilitate the functional investigation of large neuronal populations with increased SNR and accuracy in detecting the discharge of single and few action potentials.

**Supplementary Information:**

The online version contains supplementary material available at 10.1186/s40708-022-00166-4.

## Introduction

Information is encoded in the brain in the form of the coordinated activity of large populations of neurons with remarkably fine spatio-temporal resolution [1–5]. In particular, both single cell features, such as the identity of each neuron and the timing of small number of spikes or even of individual spikes [6–16], as well as the functional correlations among neurons [14, 16–20], are relevant for sensory information encoding and perception [21, 22]. Furthermore, the information encoding properties of populations of neurons are fully revealed only when analysing large populations [23, 24]. Thus, to understand how neural networks encode and transmit information it is essential to record from large populations of neurons ideally with single cell and single action potential resolution [25].

Since its introduction in neuroscience, two-photon (2P) calcium imaging [26, 27] has become one of the preferred techniques to collect data from populations of neurons, because it allows recording activity from hundreds (up to several thousands) of genetically identified neurons [11, 23, 28, 29] with subcellular resolution [30–32]. Furthermore, 2P calcium imaging is suitable for long-term studies, since cells can be tracked across different experimental sessions in longitudinal experiments [11, 33]. However, the acquisition rates of many 2P calcium imaging systems using the galvanometric mirror-based raster scanning modality result in a relatively slow temporal resolution, that does not allow fully capture calcium dynamics and fully realize the potential offered by 2P calcium imaging [34]. Another important limitation is related to the signal-to-noise ratio (SNR) of the fluorescence signal, which depends on several experimental parameters, such as reporter type and expression level, excitation laser power, local tissue properties, optical and electronic hardware, and acquisition parameters [35]. Recently developed genetically encoded calcium indicators (GECI) are characterized by excellent SNR and they achieve high accuracy in single action potential detection [36]. However, they do so under optimized experimental conditions that cannot be easily extended to large population imaging in the conventional raster scanning modality [37, 38]. To address this limitation, we [38] recently introduced Smart Line Scanning (SLS), an approach to image populations of neurons with improved temporal resolution and high signal quality. SLS combines the line scan imaging approach [39, 40] with a “smart” pixel selection strategy based on the optimization of the SNR of individual regions of interest (ROIs). SLS achieves higher imaging sampling rates, increased SNR, and larger detection accuracy of individual spikes compared with conventional raster scanning approaches when applied to neuronal population imaging.

As for conventional raster scanning, SLS requires a sequence of data processing steps to infer action potentials from the recorded fluorescence signals. For conventional raster scanning a number of tools have been developed to perform the various data processing stages (e.g., motion correction and frame registration [41–45], ROI segmentation [46–54], background subtraction [50], neuropil decontamination [55, 56], calcium activity deconvolution [50, 57–60]) and efficient toolboxes and libraries are available to pre-process 2P calcium imaging data sets [52, 61–66]. While some of the pre-processing strategies and tools developed for raster scanning data are applicable also to process SLS data (e.g., for calcium activity deconvolution), motion correction and neuropil subtraction in SLS data set specific challenges, which require the conceptual development and implementation of novel dedicated strategies. While we experimentally validated the effectiveness of SLS in our recent work [38], we did not provide a user-friendly optimized algorithm for the generation and analysis of SLS data sets in our previous work. The lack of standardized open-source available processing tools is a limitation preventing the community to benefit from the advantages of SLS.

Here, we introduce SmaRT2P, an open-source standalone and ready-to-use Matlab-based interface for the generation of SLS trajectories and the offline processing of SLS data. The SmaRT2P interface processes conventional raster acquisitions using existing standardised algorithms [44, 50], builds SLS trajectories based on reference images acquired with raster scanning, and introduces novel dedicated methods for processing SLS data. In particular, SLS trajectories are determined using a genetic algorithm to find the optimal path connecting all ROIs and the shortest path within each ROI. Furthermore, a surround region can be added to the ROIs to ensure robustness to artefacts and improve the performance of the processing algorithm. The interface allows the detection of large motion artefacts, the extraction of activity time series for each ROI, the correction of small local motion artefacts, and the reduction of background/neuropil signal from ROI traces. The motion correction algorithms, which we implemented in SmaRT2P, are line-by-line strategies specifically designed for local small motion artefacts detected in SLS data, which represent SLS-specific alternatives to the frame-by-frame motion corrections for raster acquisitions. They include the method (based on a pixels selection criterion aimed at maximizing the signal SNR) already implemented in [38] and a novel strategy, based on an efficient state-of-the art motion correction algorithm for raster data [44]. With respect to our previous implementation of SLS [38], SmaRT2P thus integrates the processing pipeline with the novel motion correction algorithm, introduces flexibility in the application of the processing steps and evaluates the processing quality using two different metrics, providing a user-friendly software for a complete processing pipeline for SLS data. We describe SmaRT2P in detail and we validate it by systematically applying SmaRT2P to a large data set of spontaneous or stimulus driven neural activity collected from head-fixed anesthetized or awake mice, which we make publicly available [67] to support further development, validation, and benchmarking of related algorithms.

## Materials and methods

### General overview

**Fig. 1:**
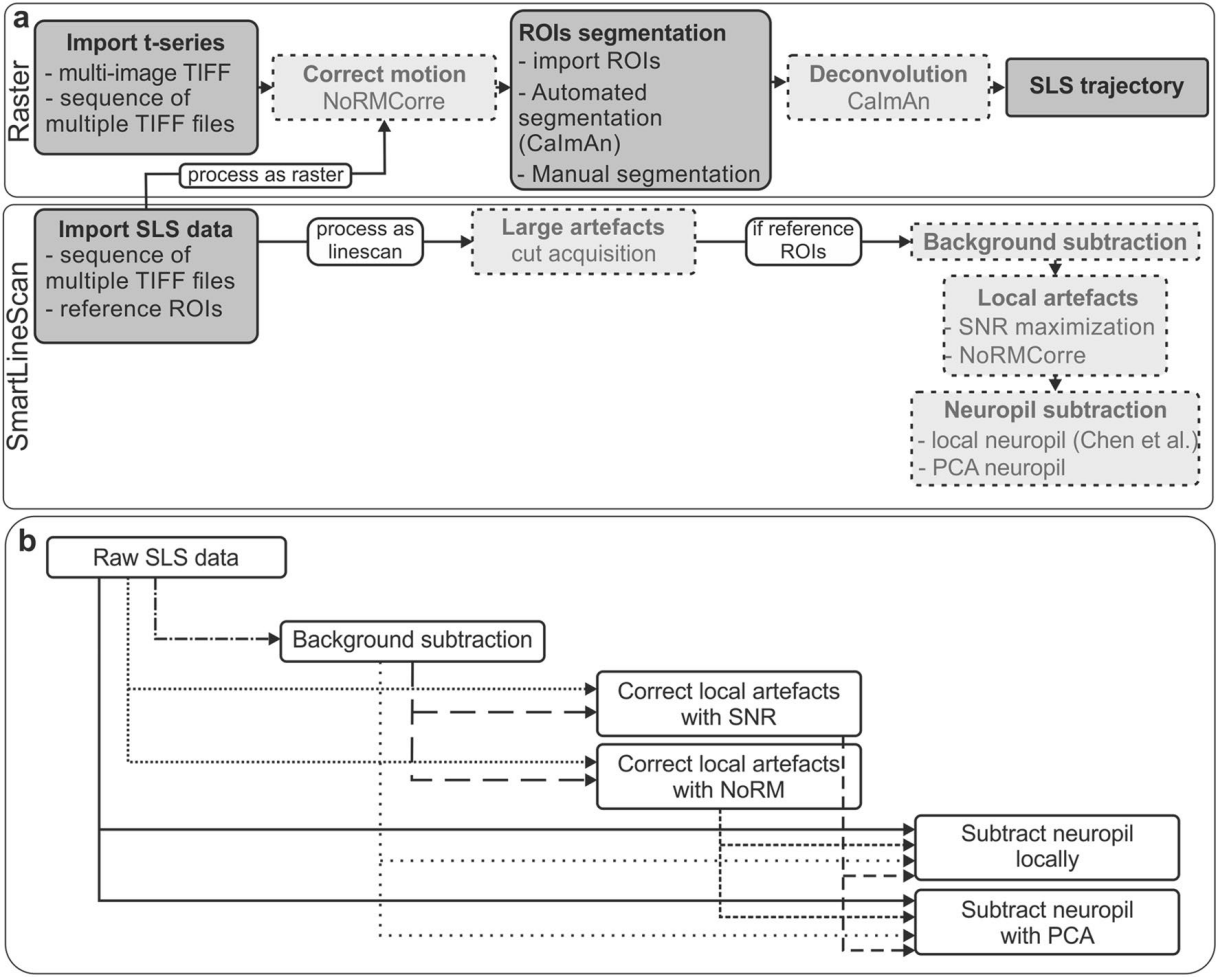
Schematic of the software.** a** Software is designed to process raster (top) and SLS (bottom) acquisitions. Dashed lighter boxes represent optional processing steps. **b** Pipeline for SLS data processing is flexible and can consist of one or many processing steps

SmaRT2P is a Matlab (R2019a) toolbox to design and process SLS acquisitions. It is composed by two main blocks (Fig. 1a): the processing of conventional raster acquisitions and building of SLS trajectories; and the processing of SLS acquisitions.

While the processing of raster data mostly relies on existing algorithms, the possibility to design SLS trajectories and to process SLS data represents the novelty of the software. The SLS processing pipeline implementation is highly flexible (Fig. 1b) to adapt to a wide range of acquisition conditions (e.g., in anesthetized or awake mice, for spontaneous or stimulus driven activity) and data quality. The presence of an intuitive user-friendly graphical interface (GUI, Additional file 1: Figure S1a) makes SmaRT2P available to users with no or basic programming experience.

Detailed information about SmaRT2P, a practical user guide and code is available at https://github.com/moni90/SmART2P.

### Raster acquisitions

#### Data import

SmaRT2P supports importing raw raster acquisitions either from a sequence of.tiff files (each file corresponding to a single frame) or from a single.tiff file (one file containing multiple images). This is a format widely used for 2P-imaging experiments, and widely used open-source software (e.g., ImageJ/FIJI [68, 69]) can convert to.tiff imaging data acquired in other popular formats.

Together with the raster data, the user must provide a series of parameters specifying the spatial and temporal resolution of the acquisition: pixel size (μm per pixel), single frame imaging period (s), single line imaging period (s) and dwell time (μs). Users can insert these parameters manually or load them from metadata stored during the acquisition. The current version of SmaRT2P allows extracting metadata from.xml documents saved by the Prairieview acquisition software (version 5.4, Ultima II scanhead, Bruker Corporation, Milan) or directly from the.tiff files recorded using ScanImage (version 2018b) [70].

Users can also import already processed raster acquisitions (previously exported from the SmaRT2P GUI and saved as Matlab files (.mat)). Thanks to the modularity of SmaRT2P, users can easily customize the interface to allow importing different data formats.

#### Motion artefacts correction

Efficient algorithms for the correction of motion artefacts are available for raster data [41–45]. We integrated in SmaRT2P a state-of-the art motion correction algorithm [44]. Users can choose between a rigid motion correction, that shifts the entire field of view (FOV) and a non-rigid motion correction, that splits the FOV in multiple patches and shifts each patch separately. The template to which frames are registered is the temporal average of the first acquisition frames. The number of frames used to compute the template as well as other parameters for the motion correction can be manually set from the GUI.

#### ROIs segmentation

For the segmentation of ROIs in the FOV, SmaRT2P provides three options: importing existing ROIs, automatically detecting ROIs or manually drawing ROIs. The segmentation obtained with any of these options can be further modified manually by users.

*Import ROIs.* Our software allows importing ROIs in various ways.

ROIs can be imported from segmentations performed in ImageJ/FIJI: ROIs from ImageJ must have been drawn using the ROI Manager tool and saved as.roi or.zip files. They are imported in SmaRT2P using the code provided in [71] and are overlapped to the FOV.

ROIs can also be imported from segmentations performed with other software/libraries: ROIs segmented using other software or libraries must be organized in a matrix of size [number of FOV pixels]x[number of ROIs], where each column corresponds to a ROI and has non-negative values in the rows corresponding to the ROI’s pixels, as in [50] and saved in.csv or.txt format. Imported ROIs are overlapped to the FOV.

Finally, ROIs can be imported from other FOVs already processed with SmaRT2P. In the case of longitudinal imaging sessions, ROIs imported from previously processed raster acquisitions on a given FOV (in the following indicated as original FOV) can be automatically adjusted to fit the current imaging session on the same area through rigid shifts and rotations. To perform the adjustment, the correlation projection of the current FOV is registered to the correlation projection of the original FOV. The transformation that minimizes the mean square error between the FOVs projections is then applied to the ROIs segmented on the original FOV and the transformed ROIs are overlapped to the current FOV. When importing ROIs, users are asked whether the automated adjustment must be performed. After the automated adjustment, users can visualize the aligned ROIs and choose whether they want to import the aligned ROIs or the original (not aligned) ones. Users can further edit the imported ROIs by adding or removing ROIs as described in the *Manual Segmentation* paragraph.

*Automated segmentation.* We integrated in SmaRT2P the possibility to perform an automated segmentation of the FOV adapting the algorithms and the codes provided in the Matlab implementation of CaImAn [61]. To segment the FOV, users are asked to manually set a putative number of ROIs and their size.

*SNR maximization criterion*. After ROIs have been imported or automatically segmented, users can further filter the pixels belonging to each ROI according to an SNR-maximization criterion. This allows reducing the number of segmented pixels and increasing the signal quality. SNR is defined as

1 $$SNR\left(f;x\right)=\frac{\underset{t}{max}f\left(t;x\right)-mea{n}_{t}\left({f}_{25}\left(t;x\right)\right)}{st{d}_{t}\left({f}_{25}\left(t;x\right)\right)},$$

where $f\left(t;x\right)$ denotes the fluorescence extracted at time $t$ and averaged across pixels $x=\left[{x}_{1},{x}_{2},\dots ,{x}_{N}\right]$ and ${f}_{25}\left(t;x\right)$ denotes all the fluorescence values below the 25^th^ percentile of the fluorescence distribution extracted from the same pixels. For each ROI, pixels are sorted according to decreasing SNR. The SNR of the ROI fluorescence trace is computed as a function of increasing number of pixels (starting with highest SNR pixels) and only pixels maximizing ROI SNR are included in the final segmentation. This SNR optimization step is performed independently for each cell in the FOV resulting in a complete segmentation mask to be used for the generation of the SLS scan path.

*Manual segmentation.* Alternatively, users can manually segment raster acquisitions in a dedicated window (Additional file 1: Fig. S1b). Users can visualize the average projection, the max–min projection and the correlation projection of the full acquisition, or alternatively visualize the average projection and the max–min projection of a small number of frames (set manually by users). Alternatively, users can browse through the acquisition, scrolling consecutive frames. Users can draw and visualize ROIs both on individual raster frames and on the projections. Each ROI can be segmented by manually drawing the contour or by selecting a bounding box. If the contour is manually drawn, an ellipse is fitted to the drawn shape. Pixels falling within the ellipse or within the bounding box are sorted for decreasing SNR [Eq. (1)] and the same SNR-maximization criterion described in the *SNR maximization criterion* paragraph is used to assign pixels to the ROI. After the automated pixels assignment, users can manually adjust the pixels selection. After a first segmentation, users can modify the segmentation by adding new ROIs or removing existing ones.

#### Calcium activity deconvolution

For the segmented ROIs, the raw fluorescence trace is computed as the average fluorescence across pixels belonging to each ROI. The normalized fluorescence is then computed as

2 $$dF/{F}_{0}\left(t\right)=\frac{f\left(t\right)-mea{n}_{t}\left({f}_{50}\left(t\right)\right)}{mea{n}_{t}\left({f}_{50}\left(t\right)\right)},$$

where ${f}_{50}\left(t\right)$ denotes all the fluorescence values below the 50^th^ percentile (or median) of the fluorescence distribution. For automatically detected ROIs, the deconvolved calcium activity is automatically extracted during the segmentation [64]. For ROIs imported or segmented manually, the same algorithm is implemented to extract the deconvolved calcium activity. The normalized fluorescence is fitted with an autoregressive model of first order in case the imaging acquisition rate is lower than 2 Hz, of second-order otherwise.

#### Data export

Processed data can be exported and saved as Matlab.mat files. A single data structure is saved with all the processing information. The same structure can be imported in the interface for further processing of the data.

#### SLS trajectory computation

Starting from a raster segmentation, the software allows drawing a SLS trajectory that travels through all the segmented ROIs (Additional file 1: Fig. S1c). The trajectory is computed to intercept each segmented pixel inside a ROI then to move to the next ROI. Among all the possible trajectories connecting segmented pixels and ROIs, SmaRT2P produces a path with minimal length inside each ROI and between ROIs [38]. We formulated the problem of finding the path with minimal length crossing all ROIs as an application of the Travelling Salesman Problem (TSP). The TSP is known to be an NP-hard problem whose brute-force solution implies to compute (n − 1)!/2 permutations and becomes computationally unfeasible for a large n, where n denotes the number of ROIs [72]. To solve the problem an approximate solution was computed, based on a genetic algorithm with a population of 100 individuals and 1000 generations. To create the next generation from the current one, the following mutations are applied to selected individuals: flipping, swapping and sliding. This allowed to generate SLS trajectories in less than 10 s. The pseudocode of the algorithm used to generate SLS trajectories is reported in Table 1.

**Table 1:** Pseudocode for SLS trajectories optimization and generation

**Input**: random path through all ROIs (*path*_*0*_), labels indicating which ROI corresponds to each *path*_*0*_ pixel (*tags*)
Step 1. Find unique ROIs
**For** ROI in unique(tags): Find ROI centroid
Step 2. Find optimal path through ROIs centroids (*path*_*TSP*_) applying genetic algorithm to solve TSP
Randomly initialize a population of 100 individuals (random paths through all ROIs) Find minimum path length *min*_*glob*_ and the individual than minimize path length (*path*_*TSP*_) **For** generation in [1:1000]: Compute the path length for each individual ** If** exist a path shorter **then** *min*_*glob*_, then update *min*_*glob*_ and *path*_*TSP*_ Randomly split population in groups of 4 individuals ** For** each group of 4 individuals: Find the individual with shortest path Create 4 mutations (original, flip, swap, slide) of the individual for the next generation
Step 3. Compute *path*_*SLS*_
Initialize empty trajectory *path*_*SLS*_
**For** ROI in *path*_*TSP*_: Find path through all ROI’s pixels (*path*_*ROI*_) using a greedy algorithm Append *path*_*ROI*_ to *path*_*TSP*_
**Return**: *path*_*TSP*_

For robustness purpose, users can add a surround of an arbitrary number of pixels around each ROIs and a reference box of arbitrary size at the end of the trajectory. ROIs mask and the SLS trajectory are exported automatically after the drawing of the trajectory: ROIs are saved as a.mat file, while trajectories are saved as an.xml file compatible with the Prairieview software (version 5.4), as a Matlab.m file compatible with ScanImage (version 2018b) and as a Matlab.mat file.

### SLS acquisitions

#### Data import and ROIs registration

SmaRT2P allows importing SLS acquisitions recorded using Prairieview (version 5.4) or ScanImage (version 2018b).

SLS data acquired with Prairieview are stored as a sequence of.tiff files, each of them containing multiple acquisitions of the SLS trajectory. When importing the acquisition in SmaRT2P, users can select the number of.tiff (from one to the total number of files set for the acquisition) which will be concatenated. SLS acquisitions can also be imported as raster acquisitions. Each SLS line is replaced by a two-dimensional FOV with same size of the original raster acquisition, whose pixels contain either the recorded fluorescence or gaussian noise (with mean and standard deviation estimated from the fluorescence of SLS trajectory pixels), depending on whether the pixel belongs to the SLS trajectory or not. If SLS data are imported as raster, only a maximum of two.tiff files can be concatenated to avoid problems due to large data structures. SLS acquisitions imported as raster can be later processed as raster acquisitions. Together with the.tiff files the.xml file containing metadata and generated during the imaging must be provided.

SLS data acquired with ScanImage are stored in a.txt file, containing metadata and information about the acquisition, and a.dat file, containing the fluorescence values. Both files should be provided to SmaRT2P.

**Fig. 2:**
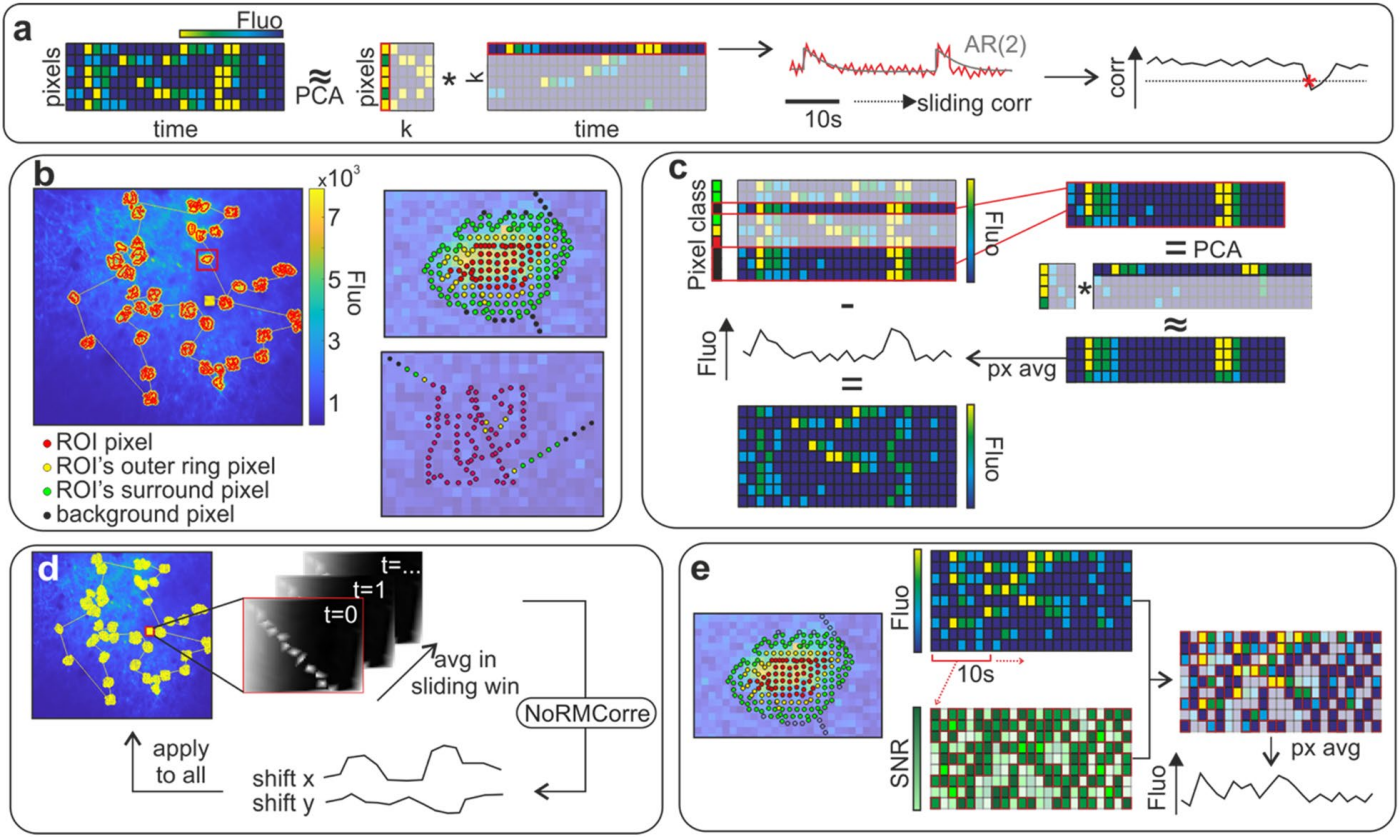
Schematic of the processing algorithms. **a** Schematic of the algorithm for large artefacts detection. The dimensionality of SLS data is reduced using PCA and the PC1 scores vector is fitted using an autoregressive model of second order (AR(2)). If the sliding correlation (computed in a 10 s window) between the PC1 scores vector and its fit drops below a given threshold (set to 0.3), a large artefact is detected (red star). **b** Left: an SLS trajectory (yellow line) with a surround of 4 pixels is overlapped to a projection of the corresponding raster acquisition and its reference segmentation (red ROIs). A reference box (yellow square) is scanned at the end of the SLS trajectory. Right top. Pixels assignment for an example ROI (red box in left panel). Pixels can be assigned to the ROI (red dots), to its outer ring (yellow dots), to its surround (green dots), or to background (black dots). Right bottom. Pixels assignment for an example ROI of a SLS trajectory without ROIs surround. **c** Schematic of the algorithm for background activity subtraction. A 1-rank representation of the fluorescence activity of trajectory pixels labelled as background (black pixels) is computed using PCA. The across-pixels averaged low-rank representation is considered as a proxy of background activity, multiplied by 0.7 and subtracted from the fluorescence of all the pixels. **d** Schematic of the small and local artefacts correction algorithm based on NoRMCorre. Activity recorded from the reference box (optionally smoothed in time by averaging in a sliding window of arbitrary width) is considered as a raster acquisition to estimate planar displacement using the NoRMCorre algorithm [44]. The estimated displacements are then applied back to the full trajectory. **e** Schematic of the small and local artefacts correction based on SNR. For each ROI, pixels labelled as belonging to the ROI, its outer ring or its surround are considered. In a sliding window of 10 s the SNR of each pixel is computed, pixels are sorted for decreasing SNR, and only a fixed number of high-SNR pixels is used to extract the fluorescence activity. Pixels are selected line-by-line considering the SNR computed in the subsequent 10 s window

During the import of a SLS acquisition, users can select the reference segmentation, that is the segmentation used to build the trajectory. In case a reference segmentation is selected, each pixel in the SLS trajectory is automatically assigned to one of the following classes according to its distance from the reference segmentation (Fig. 2b): (i) ROI: trajectory pixels within one pixel distance from the reference ROI (ii) ROI’s outer ring: trajectory pixels whose distance from the reference ROI is between one and two pixels (iii) ROI’s surround: trajectory pixels whose distance from the reference ROI is between two and four pixels (iv) Background: trajectory pixels whose distance from any reference ROI is larger than four pixels.

Pixels falling in more than one group are not assigned to any class and are discarded for further processing. The availability of a reference segmentation is not required to import SLS data but is necessary for pixels classification and for most of the following processing steps (background activity subtraction, local artefacts correction, local neuropil subtraction).

#### Large artefacts detection

The first step of the SLS pre-processing pipeline consists in the detection of large motion artefacts. These are defined as FOV displacements occurring during the acquisition associated with a shift of the ROIs away from the scanned area/trajectory. Under the assumption that such motion artefacts affect the entire SLS acquisition producing sudden (and not physiological) signal transients in all trajectory pixels, we devised the following strategy to detect a large motion artefact (Fig. 2a). We performed principal component analyses (PCA) to reduce the dimensionality of the SLS acquisition and fitted the score vector of the first principal component (PC1) with an autoregressive model of second order [50, 59] commonly used to model calcium activity. If the correlation between the PC1 scores vector and its fit, computed in a sliding window of 10 s width, drops below a threshold of 0.3 at a given timepoint, a large artefact is detected and all the data acquired after the threshold crossing are discarded.

#### Background activity subtraction

Pixels assigned to the background are used to estimate contamination of ROIs activity (Fig. 2c). The background subtraction strategy implemented here resembles the background estimate performed in [50], where pixels not segmented as ROIs were used to compute a global background activity. We considered the fluorescence activity of pixels labelled as background and we applied PCA to compute a low-rank representation of their activity (we kept only PC1, rank = 1). We then averaged across pixels the background low-rank representation, multiplied it by 0.7 [36] and subtracted it from all the SLS pixels activity. Potential negative values stemming from this procedure were set to zero.

#### Small and local motion artefacts correction

The algorithm implemented for large artefacts detection is not suited to deal with small (i.e., global events of moderate magnitude not fulfilling conditions in the *Large artefacts detection* paragraph) or local motion artefacts, defined as FOV displacements that do not cause a global misalignment between the imaged tissue and the SLS trajectory, do not result in sudden changes in fluorescence dynamics, but might nevertheless be detrimental for the quality of the extracted signal. We, therefore, implemented two strategies for the line-by-line correction of small and local motion artefacts.

The first strategy requires the user to define a reference box at the end on the SLS trajectory, ideally containing clear anatomical features (Fig. 2d). The fluorescence activity recorded from the reference box is processed as a canonical raster acquisition and the algorithm NoRMCorre [44] is applied to estimate the planar displacement of the small portion of tissue corresponding to the reference box. Users can perform a temporal smoothing (and optional down-sampling) of the reference box activity before the application of NoRMCorre, by averaging across multiple “frames” (the temporal window for smoothing and down-sampling is manually set by users). The estimated displacement is then applied to each ROI patch of the smoothed and optionally down-sampled SLS trajectory (i.e., the patch composed by pixels labelled as belonging to a ROI, including up to its outer ring and its surround).

The second strategy consists in the local reassignment of pixels based on the SNR of the extracted signal (Fig. 2e). For each ROI, pixels labelled as belonging to the ROI, to its outer ring and to its surround are pooled together. The algorithm computes, in a 10 s sliding window, the SNR of each single pixel, sorts the pixels by descending SNR and relabels as belonging to the ROI only the pixels with highest SNR. The number of pixels assigned to each ROI is set to the number of pixels belonging to that ROI in the reference segmentation. However, the identity of the pixels assigned to a ROI can vary in time as a result of small motion artefacts or displacements.

#### Neuropil decontamination

We implemented two strategies for neuropil decontamination. The first method implements the algorithm of [36]. Pixels in the surround of each ROI are used to estimate the neuropil activity. The average value of fluorescence intensity in the pixels belonging to the surround region is then multiplied by a contamination ratio of 0.7, estimated in [36], and subtracted from the average value of pixels belonging to the ROI. If pixels have been reassigned to correct local artefacts, then all the not-assigned pixels (that are variable in time) are used to compute the local neuropil. The second strategy estimates a global neuropil signal based on ROIs activity. In detail, a fluorescence signal is extracted for each ROI by averaging the fluorescence across all pixels assigned to the ROI and to its surround. Then, PCA is applied to compute a low-rank (rank = 1) representation of the ROIs activity, interpretable as a neuropil global signal. This neuropil global signal is finally subtracted from each ROI’s activity and potential negative values stemming from this procedure are set to zero.

#### Deconvolution

The fluorescence activity of each ROI is computed by averaging the processed fluorescence activity (i.e., the fluorescence after background subtraction and/or neuropil decontamination) across all pixels labelled as belonging to the ROI at the end of the processing pipeline. Deconvolution of this signal to estimate spike rates is then performed by fitting an autoregressive model of second order [64].

#### Processing pipeline: validation metrics

To quantify the results obtained with our software on real neural data, we considered two measures: the SNR of the fluorescence traces and the average pairwise correlations. Since these values vary across ROIs, for each acquisition we computed these values at fixed ROI (or ROI pair) and then we averaged across all ROIs (or ROI pairs). The reported results are the average and standard error of the mean (SEM) across acquisitions.

## Results

To demonstrate the features and performance of our software, here we present the results of processing SLS acquisitions of real 2P calcium imaging data using SmaRT2P. The data used for this purpose were recorded in mice expressing GCaMP6s in layer IV of barrel cortex (depth ~ 400 μm), using methods fully described in [38]. In brief, we used a 2P microscope equipped with galvanometric mirrors to perform raster and SLS acquisitions in anesthetized or awake head-restrained animals. In both conditions, recordings were performed both during spontaneous and sensory-evoked activity (air puff stimulation of the whisker pad). We made these data publicly available [67] to enable further tests and validations of this software and of new developments.

We first used SmaRT2P to manually segment reference raster acquisitions and compute SLS trajectories. Then, we acquired SLS data and characterized how the extracted signals are affected by each step of the SLS processing pipeline and how the SLS trajectory features (see below) impact on the quality of extracted signals. We further quantified how the SNR and pairwise correlations of ROIs depended on the processing steps. We reasoned that an effective scanning and analysis pipeline should increase (or at least maintain) the SNR as well as reduce (or at least maintain) the average level of pairwise correlations (too high values of correlations could reflect an incomplete removal or artificial covariation factors due to contaminations from global fluorescence fluctuations and neuropil activity [34, 46, 65]).

The availability of a single stand-alone toolbox to process raster and SLS data allowed us to easily switch between the raster and the line scan modalities in the same experimental session. This is especially useful for long experimental sessions, where some displacement of the FOV across different recordings might occur. In this latter case, a possible strategy is to alternate SLS acquisitions with short raster acquisitions, align the initial raster segmentation to the novel raster acquisitions (keeping the same ROIs but correcting for possible displacements), and recompute SLS trajectory over time according to the movements recorded by the alternating raster acquisitions. Since ROIs previously segmented using SmaRT2P are automatically imported and aligned to the raster FOV, this procedure contributes to generating more robust SLS data at small cost.

### Detection of large motion artefacts

The effect of planar motion artefacts can be very different between raster scan and SLS. In raster scan acquisitions, an image retaining all topological features of the FOV is usually available. Thus, planar motion artefacts can be compensated for with a frame-by frame iterative process, using a reference image of the FOV and a rigid or non-rigid approach. These strategies are in general efficient against moderate artefacts for most of the ROIs [44, 62]. In SLS acquisition, planar displacements of the FOV of size comparable to a pixel can cause a mismatch of cellular structures with respect to the set of pixels selected for the scanning trajectory. Consequently, the set of pixels in each ROI scanned by SLS might no longer be optimized based on the collective SNR of the ROIs. In case of large motion artefacts (> 5 µm), this displacement can be large enough to exclude the cells of interest from the SLS trajectory, in particular when no ROIs surround is included [38]. Since a complete bidimensional image of the cells of interest is lacking in SLS, we cannot use topological features of the acquisition nor a reference FOV image to estimate the planar displacement. We thus defined large motion artefacts based on functional features, ensuring that the recorded signals otherwise fluctuate smoothly in time and follow dynamics that are characteristic of calcium activity (see “Large artefacts detection” section). In our experimental setup, large artefacts were seldom detected in SLS acquisitions in anesthetized animals (num. detected artefacts = 16 out of n = 258 SLS acquisitions from n = 22 mice), while they were more frequent in awake and running head-restrained animals (num. detected artefacts = 7 out of n = 28 SLS acquisitions from n = 4 mice).

**Fig. 3:**
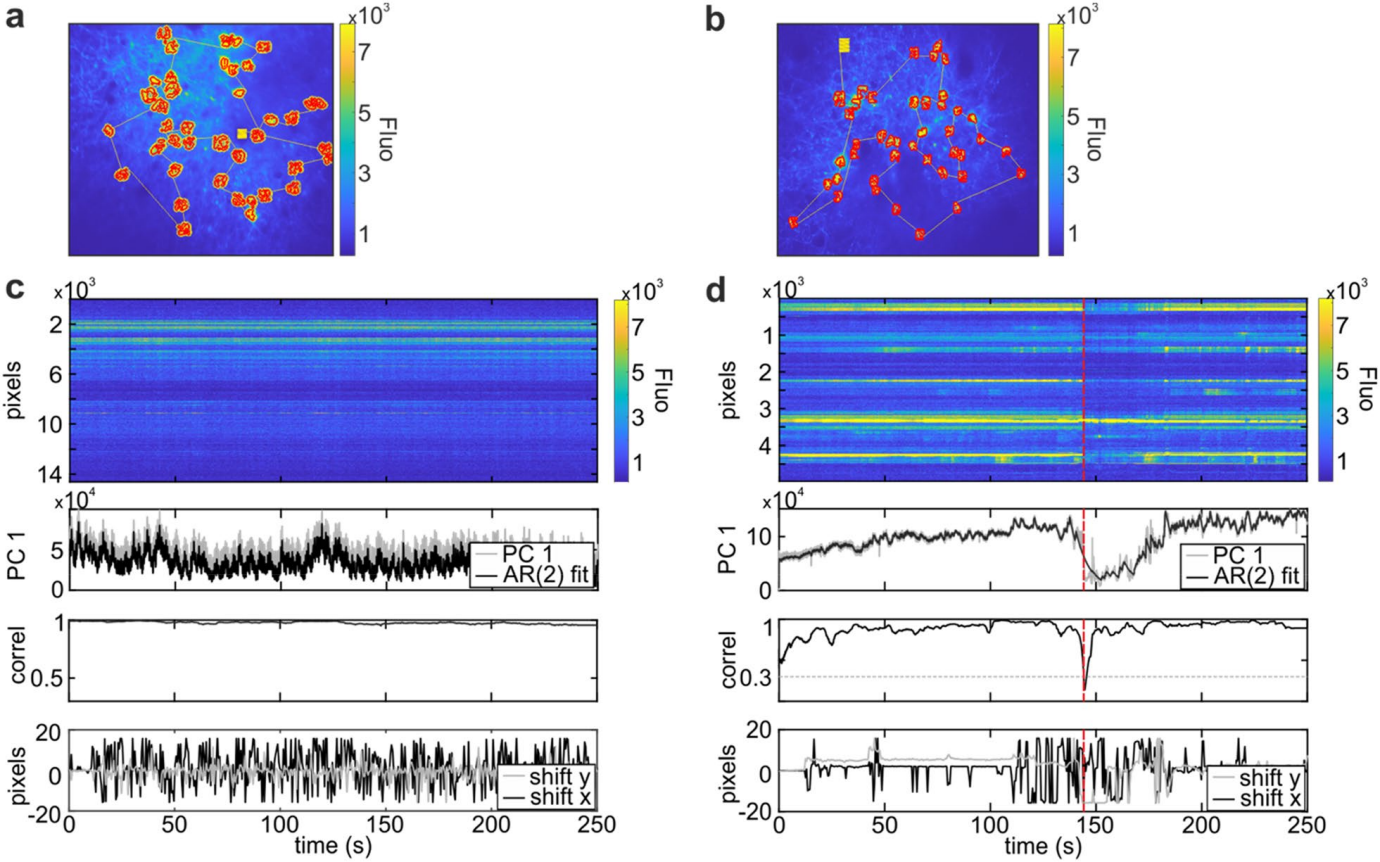
Large artefacts detection.** a**, **b** SLS trajectories (yellow line) with a surround of 4 pixels (**a**) and without surround (**b**) are overlapped to a projection of the corresponding raster acquisition and to their reference segmentation (red ROIs). **c**, **d** Large artefact detection for the acquisition in **a**, **b**. From top to bottom. Raster of the SLS acquisition. Each row represents the fluorescence trace of a pixel in the trajectory. Pixels are ordered according to their position in the trajectory, from the first (top) to the last (bottom). Reduced representation of SLS dynamics using only the scores vector of the first PC (PC 1, grey line) and fit with an AR(2) model (black line). Correlation between the first PC scores vector and its fit. Displacement along the horizontal (black) and vertical (grey) direction estimated applying the NoRMCorre algorithm [44] to the reference box. The dashed red line in **d** denotes the detection of a large artefacts

We investigated whether the presence and the size of a surround (i.e., the inclusion in the SLS of a region surrounding the ROI identified based on pixel SNR; surround dimension: 1, 2, 3 pixels) improved the robustness of SLS acquisitions to large motion artefacts (Fig. 3). We restricted the analyses to SLS data from awake animals, where detected large artefacts were less rare and a reference box (see “SLS trajectory computation” section) at the end of the SLS trajectory was always available. We considered the reference box pixels as a raster acquisition and we applied the NoRMCorre algorithm [44] to this small FOV portion to obtain an estimate of the amount of planar displacement of the FOV. We then estimated the fraction of SLS acquisition discarded *a-posteriori* using a linear mixed model in which the size (in pixels) of the ROI surround ($p{x}_{surr}$) was treated as a fixed effect covariate, and the estimated planar displacement was treated as a random effect:

3 $$\frac{discardedSLSlines}{acquiredSLSlines}=\left({\beta }_{0}+{\beta }_{1}*p{x}_{surr}\right)+\left({\beta }_{0,shift}+{\beta }_{1,shift}*p{x}_{surr}|shift\right)+\varepsilon$$

This model distinguishes the specific contribution of the SLS surround size on the large motion artefact detection (${\beta }_{0}+{\beta }_{1}*p{x}_{surr}$) from the contribution due to the effective FOV displacement, estimated using the NoRMCorre algorithm (${\beta }_{0,shift}+{\beta }_{1,shift}*p{x}_{surr}|shift$). We expect that larger planar displacements in the acquired FOV will increase the fraction of discarded lines and we aim to understand whether adding a surround has any effect on the amount of discarded data. We found that the part of the model capturing the effect of surround size predicted that larger surrounds led to a significant decrease in the fraction of SLS acquisition to discard (${\beta }_{1}=-0.08$, *p* value = 0.036, model predictions with 95% confidence intervals and residuals q–q plot in Additional file 1: Figure S2). This suggests that SLS trajectories with surrounds are more robust to motion artefacts (Fig. 3a,c). This was confirmed by the further finding that SmaRT2P detected large motion artefacts mostly for the acquisitions without surround (Fig. 3b, d; Table 2). The increased robustness to motion artefacts was obtained at the cost of decreasing the single line sampling rate because of the larger number of pixels to scan when the surround is included (Table 2).

**Table 2:** Sampling rate and large artefacts detection. Mean ± SEM

Anesthetized				
	Surr = 0	Surr = 1	Surr = 2	Surr ≥ 3
N SLS	146	54	41	17
Sampling rate (Hz)	42.7 ± 1.6	29.2 ± 2.3	23.3 ± 2.3	18.7 ± 2.6
N SLS with large artefacts	8	5	3	0
Fraction of cropped lines	0.65 ± 0.27	0.76 ± 0.14	0.63 ± 0.27	N.A

Awake				
	Surr = 0	Surr = 1	Surr = 2	Surr ≥ 3
N SLS	16	3	2	7
Sampling rate (Hz)	29.5 ± 4.1	28.2 ± 12.1	16.1 ± 0	16.6 ± 0.2
N SLS with large artefacts	6	0	0	1
Fraction of cropped lines	0.68 ± 0.34	N.A	N.A	0.11 ± 0.00

### Background activity subtraction

**Fig. 4:**
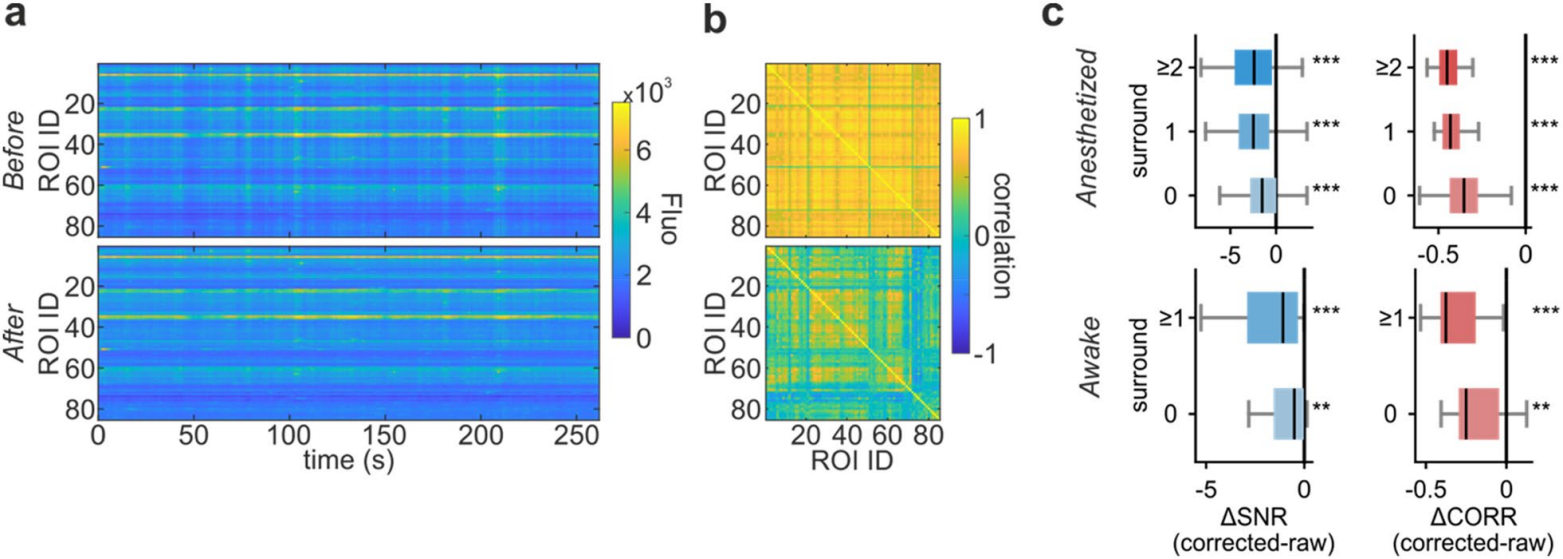
Background activity subtraction. **a** Top. Fluorescence activity of the ROIs segmented in an example SLS acquisition. Each row in the raster corresponds to the activity of a ROI. Bottom. Fluorescence activity of the same ROIs after the subtraction of the background activity. **b** Pairwise correlations between ROIs in **a** before (top) and after (bottom) the subtraction of the background activity. **c**. Average SNR (blue, left) and pairwise correlations (red, right) difference between background-subtracted and raw data for anesthetized (top) and awake animals (bottom). Extended values in Table 3

In 2P imaging experiments, it is common practice to subtract global background activity, i.e., the signal generated by neighbouring unspecified fluorescent structures [34, 50, 62]. In conventional raster scanning, global background activity can be computed from global fluctuations of the whole FOV or of FOV regions without ROIs (for example, the background components in [50]). In SLS acquisitions, a smaller number of pixels not belonging to ROIs are acquired, thus restricting the surface available for background estimation. We developed a strategy to estimate and subtract global background fluctuations using the available pixels and assessed its effectiveness by computing pairwise correlations between ROIs fluorescence traces before and after the subtraction of the global background activity (Fig. 4). A significant reduction in pairwise correlations after background activity subtraction was observed both in anesthetized and awake animals (Fig. 4b,c), suggesting that our strategy was effective in removing simultaneous fluctuations in ROIs fluorescence (Table 3).

**Table 3:** Background subtraction. Difference in pairwise correlations before and after background subtraction

	Anesthetized			Awake	
	Surr = 0	Surr = 1	Surr ≥ 2	Surr = 0	Surr ≥ 1
N SLS	146	53	57	16	12
∆ SNR	− 1.62 ± 0.21 (2.3e−13)	− 2.35 ± 0.39 (1.9e−6)	− 2.47 ± 0.37 (2.6e−7)	− 5.25 ± 4.26 (0.0023)	− 3.16 ± 1.56 (4.9e−4)
∆ pairwise correlations	− 0.35 ± 0.01 (1.0e−25)	− 0.42 ± 0.01 (2.4e−10)	− 0.43 ± 0.01 (5.1e−11)	− 0.20 ± 0.04 (0.0019)	− 0.30 ± 0.04 (0.0005)

Only acquisitions with a reference raster segmentation are considered. Mean ± SEM (and *p* values for Wilcoxon signed rank test)

### Small and local motion artefacts correction

As discussed above, rigid and non-rigid motion correction techniques developed for raster scanning data do not generalize to SLS data. We, therefore, developed two line-by-line motion correction strategies for SLS data, which compensate small or local displacements that might take place during the acquisition.

The first strategy, which is novel with respect to the algorithms presented in [38], is the SLS line-by-line analog of the rigid frame-by-frame motion correction for raster acquisitions. It can be implemented only when a reference box is available at the end of the SLS trajectory and it uses the reference box as a raster acquisition to estimate the planar displacement. Ideally, the reference box should contain clear anatomical features that remain available for all the duration of the recording, otherwise the motion estimate algorithm would try to match noise to a template and fail. Since the displacement computed in a small FOV portion is then applied back to the full trajectory, this procedure assumes that the FOV is not altered by deformations but rigidly shifts within the imaged plane. We did not explicitly verify this assumption, but we believe it could be satisfied for SLS acquisitions, because they are characterized by high sampling rates, while non-rigid deformations are in general associated with slow acquisition rates [41, 43, 44]. To make the motion correction algorithm more robust against noise, we also estimated motion using a smoothed version of the data, obtained by averaging activity in a sliding window (width = 1 and 10 s).

The second strategy, already applied in [38], has been developed *ad-hoc* for SLS acquisitions. It is implemented locally around each ROI and aims at maximizing signal quality by considering only pixels with high SNR. Such strategy allows getting a different displacement for each ROI, therefore, releasing the assumption of a rigid FOV. This algorithm provides, therefore, a line-by-line pixel reassignment for each ROI, but it integrates information from a larger time window (to compute SNR) to compute single-line corrections. It must be noted that the success of this algorithm might be limited for those ROIs characterized by long silent periods. In fact, during such periods there is no signal available to compute the SNR and the estimated displacements would be based on noise fluctuations.

**Fig. 5:**
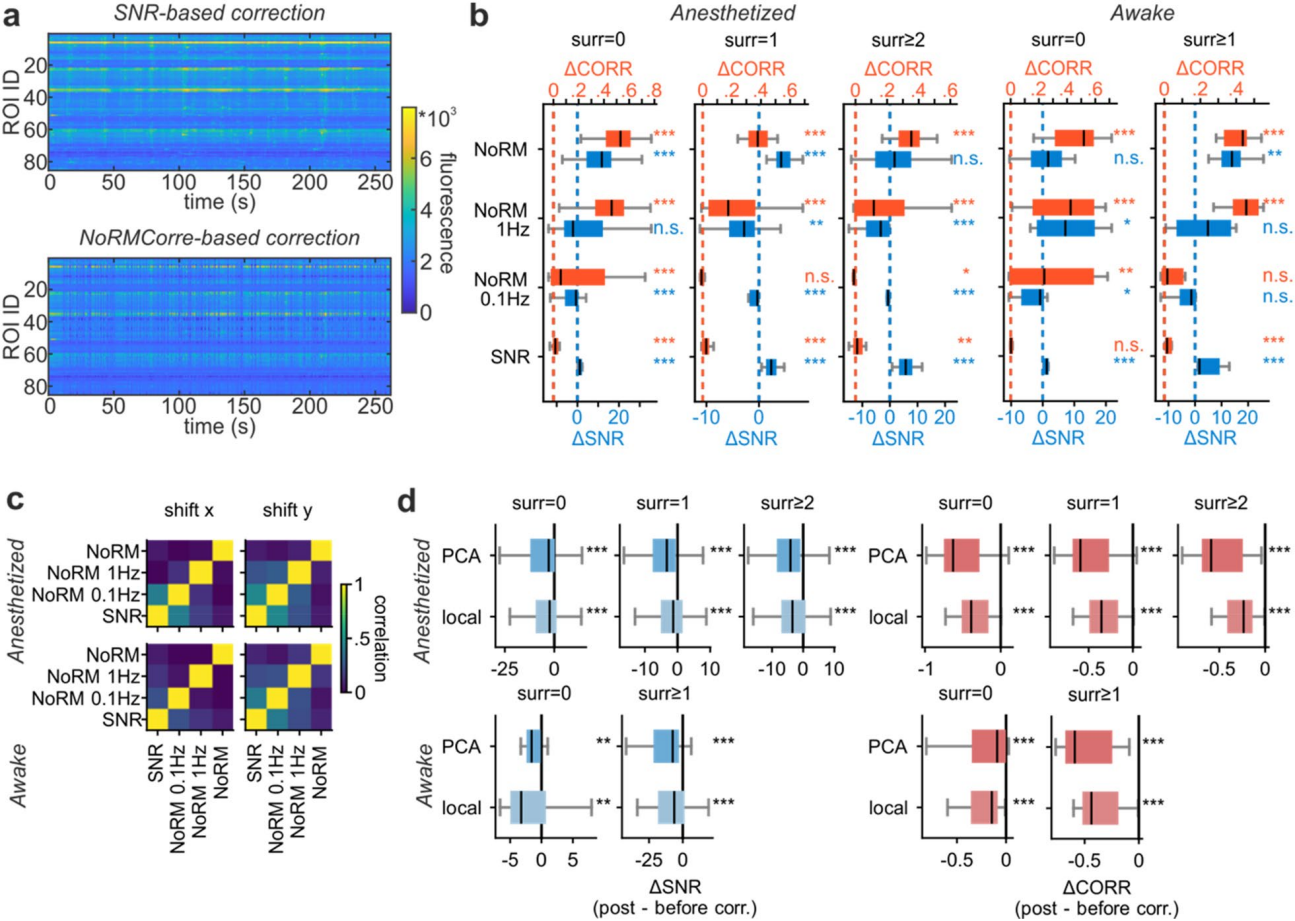
Small and local artefacts correction and neuropil decontamination. **a** Fluorescence activity of the same ROIs of Fig. 2a after the correction of artefacts obtained through the reassignment of those pixels that maximize the SNR (top) or through the correction strategy that uses the displacements estimated using the NoRMCorre algorithm [44] on the reference box with a smoothing window of 10 s (bottom). **b** Effects of different artefacts corrections strategies on the SNR and pairwise correlation of fluorescence traces for different surrounds and animal states. Extended values in Table 4. **c** Correlations between the displacement along the *x*-axis (left) or *y*-axis (right) estimated using different strategies in anesthetized (top) and awake (bottom) animals. **d** Effects of neuropil decontamination strategies on the SNR and pairwise correlation of fluorescence traces for different surrounds and animal states. Extended values in Table 5

We applied both strategies after the global background subtraction to all the SLS acquisitions, where a reference box was available and we observed that the SNR-based strategy leads to a small but consistent increase in the SNR of the extracted traces, independently from the surround size and the animal condition (Fig. 5b; Table 4) and to small or non-significant increase in the pairwise correlations between ROIs (Fig. 5b; Table 4). In general, the increase in signal quality is larger and more significant for larger surrounds, which suggests that adding surrounds to the segmented ROIs might not only increase robustness with respect to large motion artefacts but also improve the performance of the local motion artefacts correction algorithm. The strategy based on the NoRMCorre algorithm [44] had heterogeneous performance, depending on the size of the surround and the temporal smoothing. In terms of SNR, it performed better for faster acquisitions without temporal smoothing and with small or no surround (Table 4). When it was successful in increasing the SNR, the improvement was larger than the one obtained using the SNR-based strategies. However, it was also accompanied by a larger increase in pairwise correlations, which might suggest that some background activity is retained in the ROIs traces (Table 4).

**Table 4:** Small artefacts motion correction

		Anesthetized			Awake	
		Surr = 0	Surr = 1	Surr ≥ 2	Surr = 0	Surr ≥ 1
	N SLS	101	33	28	16	12
SNR	∆ SNR	1.28 ± 0.05 (3e−18)	2.39 ± 0.19 (5e−7)	5.72 ± 0.50 (4e−6)	1.07 ± 0.14 (0.0004)	4.46 ± 1.35 (0.0005)
	∆ pairwise correlations	0.016 ± 0.001 (3e−14)	0.023 ± 0.003 (4e−6)	0.016 ± 0.005 (0.0094)	0.003 ± 0.002 (0.0627)	0.028 ± 0.007 (0.0005)
NoRMCorre 0.1 Hz	∆ SNR	− 0.55 ± 1.11 (8e−7)	− 2.11 ± 0.67 (5e−7)	− 1.68 ± 0.53 (4e−6)	8.14 ± 12.61 (0.0494)	− 1.74 ± 3.79 (0.0640)
	∆ pairwise correlations	0.201 ± 0.024 (8e−8)	0.065 ± 0.029 (0.5143)	0.011 ± 0.017 (0.0256)	0.29 ± 0.07 (0.0052)	0.12 ± 0.05 (0.0923)
NoRMCorre 1 Hz	∆ SNR	3.46 ± 1.38 (0.8244)	− 1.80 ± 1.44 (0.0064)	− 3.87 ± 1.42 (7e−5)	13.86 ± 5.54 (0.0131)	4.41 ± 3.30 (0.3013)
	∆ pairwise correlations	0.430 ± 0.019 (3e-−18)	0.233 ± 0.035 (2e−6)	0.156 ± 0.034 (4e−4)	0.39 ± 0.06 (0.0004)	0.42 ± 0.04 (0.0005)
NoRMCorre	∆ SNR	11.01 ± 0.94 (2e−15)	4.81 ± 0.94 (9e−5)	1.83 ± 1.74 (0.3275)	7.44 ± 4.84 (0.2553)	12.67 ± 2.31 (0.0015)
	∆ pairwise correlations	0.521 ± 0.012 (3e−18)	0.370 ± 0.022 (5e−7)	0.321 ± 0.022 (4e−6)	0.46 ± 0.04 (0.0004)	0.41 ± 0.02 (0.0005)

Difference in SNR and pairwise correlations before and after motion correction with different methods. Only acquisitions with a reference raster segmentation and a reference box in the SLS trajectory are considered. Mean ± SEM (and *p* values for Wilcoxon signed rank test)

To further understand the different performance of the two strategies, we computed the correlation between the (planar) displacements estimated by each algorithm (Fig. 5c). In the SNR-based strategy, which is characterized by local and non-rigid artefacts correction, we defined a planar displacement for each ROI, as the planar shift of the centre of the ROI spatial footprint. We then computed correlations between each ROI’s SNR-based displacement and the NoRMCorre-based displacement and considered the maximum value of correlation across ROIs as a measure of similarity between the displacements estimated using the two strategies. We found that correlation values where higher between the SNR-based displacement and the NoRMCorre-based displacement with smoothing at 0.1 Hz (correlation values. *x*-axis: 0.42 ± 0.01, *y*-axis: 0.46 ± 0.01, mean ± SEM, n = 162 anesthetized. Correlation values. *x*-axis: 0.25 ± 0.03, *y*-axis: 0.41 ± 0.04, mean ± SEM, n = 28 awake). This might be explained by the fact that the temporal window considered for the SNR computation and the smoothing applied before the NoRMCorre-based correction have the same width. The correlation values obtained when considering smaller or no smoothing windows were smaller.

### Neuropil decontamination

Fluorescence signal from out of focus sources or from subresolved structures (i.e., neuropil) might contaminate the ROIs signals and generate false detections of calcium transients when using SLS or raster scans [11, 36, 73]. State-of-the-art techniques for neuropil decontamination in raster acquisitions provide accurate estimates of the neuropil signal by leveraging on the fluorescence extracted from pixels in each ROI surround [36, 55, 56]. In SLS acquisitions, ROIs surrounding regions are not necessarily scanned or might contain too few pixels and available strategies thus cannot be applied. We then implemented two strategies for neuropil decontamination in SLS acquisitions: a local strategy, which estimates a neuropil signal for each ROI by averaging across the available neighbouring pixels and a global strategy, which provides a single estimate of the neuropil signal using dimensionality reduction. We applied both neuropil decontamination strategies as final step of multiple processing pipelines (Fig. 1b) to all SLS acquisitions with a reference box. Pooling results together across different pipelines showed that both neuropil decontamination strategies led to significant decreases in pairwise correlations (Fig. 5d; Table 5), suggesting they are effective in reducing artificial covariations likely due to neuropil contamination.

**Table 5:** Local neuropil subtraction

		Anesthetized			Awake	
		Surr = 0	Surr = 1	Surr ≥ 2	Surr = 0	Surr ≥ 1
	N SLS	1010	330	280	120	160
Local surround	∆ SNR	9.15 ± 5.93 (4e−46)	10.45± 5.49 (6e−8)	– 3.82 ± 0.47 (8e−24)	− 4.19 ± 2.95 (7e−4)	− 1.36 ± 3.82 (4e−3)
	∆ pairwise correlations	− 0.337 ± 0.006 (2e−159)	− 0.324 ± 0.011 (9e−54)	− 0.263 ± 0.010 (1e−45)	− 0.313 ± 0.017 (5e−26)	− 0.287 ± 0.018 (2e−19)
PCA	∆ SNR	6.76 ± 0.35 (3e−75)	− 4.28 ± 0.33 (2e−32)	− 5.18 ± 0.43 (8e−28)	− 10.51 ± 2.30 (4e−13)	− 9.98 ± 1.09 (3e−11)
	∆ pairwise correlations	− 0.532 ± 0.008 (9e−160)	− 0.495 ± 0.012 (1e−53)	− 0.495 ± 0.013 (1e−45)	− 0.418 ± 0.022 (9e−26)	− 0.448 ± 0.022 (2e−19)

Difference in SNR and pairwise correlations before and after local neuropil subtraction with different methods. Only acquisitions with a reference raster segmentation and a reference box in the SLS trajectory are considered. Mean ± SEM (and *p* values for Wilcoxon signed rank test)

### Processing pipeline

**Fig. 6:**
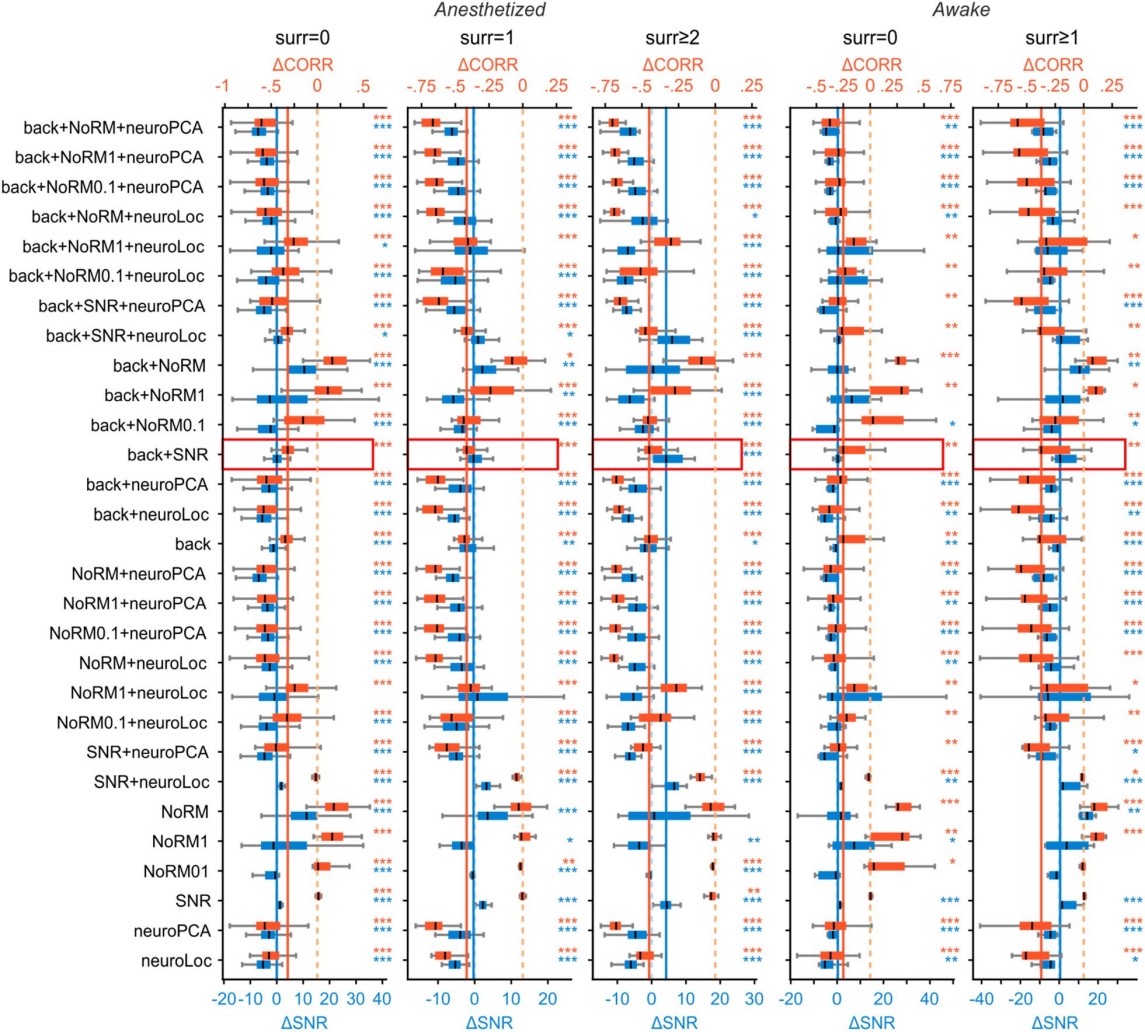
Processing pipelines comparison. Evaluation of the signal quality after different processing pipelines for SLS with different surrounds in anesthetized (n = 101, 33, 28 for surround 0,1, ≥2) and awake animals (n = 16, 12 for surround 0, ≥1). Blue boxplots show the SNR distributions for each pipeline, red boxplots show the pairwise correlations distributions for each pipeline. The continuous blue and the orange lines show, respectively, the SNR and pairwise correlation median value for the processing pipeline composed by background subtraction followed by SNR-based artefacts correction (red box). The dashed light blue and light orange lines correspond to no changes in SNR and pairwise correlations, respectively, with respect to raw data. Extended values in Additional file 1: Table S1

Since there is no standard processing for SLS acquisitions, we combined the processing steps described above in different ways and assessed the effect of the full processing pipeline measuring the changes of SNR and pairwise correlations with respect to raw data (Fig. 6). As expected, processing pipelines comprising only local corrections for signal optimization (denoted by SNR and NoRM in Fig. 6) were not effective in reducing pairwise correlations. This suggests that background and/or neuropil contamination might still be present, which advises against the choice of such processing pipeline. Most other processing pipelines, in general, resulted in a decrease in pairwise correlations associated with more or less marked decrease in SNR. For our data, we found that the pipeline composed by background subtraction followed by SNR-based local motion artefacts correction and, optionally, local neuropil decontamination was the most effective in reducing correlations with respect to the raw data while preserving signal quality (detailed values for all pipelines are reported in Additional file 1: Table S1).

## Discussion

Two-photon calcium imaging is a leading technique for functional readout of neural activity, enabling the recording of the activity of populations of neurons with high spatial information in depth and across multiple experimental sessions. New calcium indicators have been engineered [36, 74] and a wide array of software and analysis tools have been developed by the computational neuroscience community for the post-processing and advanced analysis of these data [35, 44, 51, 53, 55, 62, 64, 66]. Using these tools and under optimized imaging conditions (i.e., while imaging one or a few cells at high frame rate), the detection of the discharge of one or few action potentials in individual cells has been demonstrated [36]. However, achieving similar performance while recording large populations of neurons or at increased acquisition rates requires the improvement of scanning procedures to acquire data from the regions of interest with the highest possible speed and SNR. To this aim, we recently introduced Smart Line Scanning and we demonstrated that it improves the temporal resolution and signal quality of two-photon calcium imaging data [38], improving the accuracy of single spike detection when recording neural population activity from awake animals. Here, we complement that effort providing a novel open-source software toolbox, SmaRT2P, which greatly simplifies the implementation of smart line scanning in standard 2P microscopes. Our toolbox is provided with a range of tools for both the design and implementation of the smart line scanning, and for the data processing. The implications of our work are discussed in what follows.

### Software block for raster acquisitions

SmaRT2P is composed by two main blocks. The first block processes raster acquisitions. We added the raster data processing to SmaRT2P not only to have a stand-alone tool, but also because an initial raster scanning imaging section is needed to design the SLS, and because users may want to use either type of scanning depending on the experimental conditions or scientific questions. SmaRT2P was designed to easily allow computing SLS trajectories from raster scanning data and switching from raster acquisitions to line scan acquisitions with minimal requirements.

The raster scanning block of SmaRT2P is largely based on existing algorithms. However, and importantly, SmaRT2P adds the possibility of importing raster acquisitions, which are already segmented. In this way, users can segment raster data using their preferred and independent tool, ensuring full compatibility of SmaRT2P with raster scanning procedures already established in each laboratory. The first SmaRT2P block differs from available toolboxes for the possibility of drawing of SLS trajectories, which cross through all the ROIs identified from raster acquisitions. Before generating the SLS trajectory, the users have the option to add a surround to each ROI and to add a reference box at the end of the trajectory, that is scanned in a raster-like modality. The users can manually set the width of the surround, the size of the reference box, and its position within the FOV.

### Software block for designing and implementing SLS trajectories

Once the ROIs are segmented in the raster scanning software block, SmaRT2P optimizes the SLS trajectories in an automated way. Finding an optimal trajectory is particularly important for recordings of large populations. In this case, scanning the ROIs in a sub-optimal way might result in numerous jumps of the laser within the FOV and reduce the acquisition speed and the advantages provided by the SLS approach. However, the problem of finding the optimal trajectory is computationally hard, is well studied in the literature as the TSP, and its complexity increases steeply with the number of ROIs. We, therefore, used a genetic algorithm to compute an approximate solution instead of the optimal one. To make SLS trajectories more robust to motion artefacts and to background/neuropil contamination, ROI’s surround regions and a reference box can be included in the SLS trajectory. The presence of the surround regions and of the reference box decreases the speed of SLS acquisitions but might be particularly useful for data acquired in awake animals, where the presence of these artefacts might limit the applicability of line-scan approaches.

Importantly, the second block of SmaRT2P provides various tools for the processing and analysis of data acquired with SLS. To our knowledge, SmaRT2P represents the first open-source tool available for this type of analyses. It combines algorithms used in [38] (but not shared in an open-source toolbox) with a novel algorithm for the correction of local artefacts in SLS data. More specifically, SmaRT2P allows detecting large motion artefacts in SLS acquisitions, to subtract background and neuropil contamination, and to detect and correct small and local artefacts. The users can choose to apply one or multiple processing steps to SLS data according to their needs.

SmaRT2P is equipped with a graphical interface, which facilitates its use to people without programming experience. The graphical interface includes methods for the generation of SLS trajectories and for a complete processing of SLS data (i.e., motion artefacts detection and correction, background subtraction, and neuropil decontamination). We designed SmaRT2P to be flexible, to adapt to data acquired under different experimental conditions. A first degree of flexibility is provided in the design of the SLS trajectories, where users can set a trade-off between increased temporal resolution and robustness to motion artefacts. Then, in the SLS data processing, SmaRT2P is organized in a modular way and different processing pipelines can be implemented. We did not set a preformatted processing pipeline, because smart line scan acquisitions and smart line scan processing tools are sensitive to trajectory characteristics and to experimental conditions. The processing pipeline should then be tuned according to the features of the acquired data. Furthermore, a modular approach is open to extension, modifications, and insertion of further developments of the processing algorithms.

### Results on benchmarking SmaRT2P on real data

We benchmarked the performance of SmaRT2P on an extensive 2P GCaMP6s data set recorded from layer IV of the barrel cortex in anesthetized and awake mice. This data set was larger than the one used in [38]. We selected the SNR of the extracted signals and their average pairwise correlations as indicators of the algorithms’ performance, and we computed changes in these two measures as a function of each processing step and of different processing pipelines (i.e., combinations of multiple processing steps in a given order). Results of this benchmarking indicated that combining SLS and SmaRT2P allows successfully acquiring data with high sampling rate and high signal quality. A careful choice of few parameters in the design of the SLS trajectories (ROIs surround and reference box) improves the performance and effectiveness of the SLS processing techniques and allows reaching a trade-off between fast and robust recordings. A systematic study of the effect of variations in the processing pipeline showed how to optimize the pipeline depending on the data set type and suggested that combining a background or neuropil subtraction algorithm with an artefact correction algorithm should successfully improve the signal quality and remove global fluctuations and artefacts that might mask relevant single cell activity. Importantly, we made the benchmarking 2P data set fully available with this article, to enable open-source development of new raster scan tools and to facilitate an open and fair comparison of performance with future algorithms.

### Hardware requirements

On the hardware side, SLS acquisitions can be performed using the same setup used for conventional raster acquisition. This is possible, because SLS trajectories are mapped on the FOV acquired in raster mode and are tuned to the characteristics of the microscope used for raster acquisitions. When moving from one pixel to the adjacent one, mirrors, therefore, perform the same type of movements required for raster acquisition.

## Conclusions

The growth and flourishing of 2P calcium imaging for studying neural population activity has been paralleled by major computational effort for the development of open-source toolboxes for the analysis of such data. Thus, the development of analytical tools for 2P calcium imaging is a major frontier in current computational neuroscience [44, 45, 48, 51, 53–56, 61, 62, 64, 66, 75]. However, less attention has been given to the development of open-source toolboxes that improve the scanning and acquisition procedures. Our open-source toolbox, SmaRT2P, contributes to filling this gap and will facilitate further development in the field. Our validations of SmaRT2P on real data suggest that SmaRT2P will improve the resolution and accuracy with which neural population codes can be extracted from 2P imaging experiments.

## Supplementary Information

**Additional file 1: Figure S1**. SmaRT2P graphical user interface. a. Schematic of the main GUI window of SmaRT2P. In this window users can visualize the raster movie, the segmented ROIs, and the extracted activity. b. Schematic of the GUI window for the segmentation of raster data. Users can visualize simultaneously multiple projections of the acquisition (left) and scroll through single frames (right). Users can segment ROIs using a bounding box (red inset, left) or manually drawing the contour (red inset, right) both on the large projection window and on the sliding frames. The smart selection of the pixels includes in the segmentation only those pixels that maximize the extracted SNR (bottom right), but users can adjust the pixels selection manually. c. Example SLS trajectory (yellow line) with a small surround and a reference box attached at the end of the trajectory. Coloured areas show the ROIs used to build the trajectory. **Figure S2**. Linear mixed model. a. Prediction of the fraction of SLS acquisitions to discard as a function of the number of pixels in the ROI’s surround region (gray area represents 95% confidence intervals). The model shows that scanning larger surrounds reduces the number of SLS lines to discard adding robustness to SLS acquisitions. b. QQ plot of the residuals of the data after fitting the linear mixed model to model the fraction of discarded SLS acquisitions. Since normality is a necessary condition to apply linear mixed models, we re-fitted the model after removing one outlier and observed that results were similar to the ones obtained using the full data set (beta1 = −0.11, *p* value=0.048). **Table S1**. Mean ± SEM (and *p* values for Wilcoxon signed rank test) for SNR and pairwise correlations of different processing pipeline reported in Fig. 6.

## Data Availability

The data sets generated and/or analysed during the current study are available in the following repository: Brondi, M., Moroni, M., Panzeri, S., & Fellin, T. (2021). *Recordings of mice layer IV barrel cortex activity using two-photon functional microscopy and Smart Line Scanning* [67]. EBRAINS. https://doi.org/10.25493/74DX-JVC. Detailed information about SmaRT2P, a practical user guide and the open-source-code of the toolbox can be downloaded at https://github.com/moni90/SmART2P. The software is released under the MIT license.

## Abbreviations

2P: Two-photon
SNR: Signal-to-noise ratio
GECI: Genetically encoded calcium indicator
SLS: Smart line scan
ROI: Region of interest
GUI: Graphical user interface
FOV: Field of view
PCA: Principal component analysis
PC1: First principal component
SEM: Standard error of the mean
