# Supplementary material for: SmaRT2P: a software for generating and processing smart line recording trajectories for population two-photon calcium imaging
Source: Brain Inform. 2022 Aug 4;9(1):18. doi: 10.1186/s40708-022-00166-4 (PMC9352634; doi:10.1186/s40708-022-00166-4)
Supplement: Supplementary file 1 — Additional file 1: Figure S1. SmaRT2P graphical user interface. a. Schematic of the main GUI window of SmaRT2P. In this window users can visualize the raster movie, the segmented ROIs, and the extracted activity. b. Schematic of the GUI window for the segmentation of raster data. Users can visualize simultaneously multiple projections of the acquisition (left) and scroll through single frames (right). Users can segment ROIs using a bounding box (red inset, left) or manually drawing the contour (red inset, right) both on the large projection window and on the sliding frames. The smart selection of the pixels includes in the segmentation only those pixels that maximize the extracted SNR (bottom right), but users can adjust the pixels selection manually. c. Example SLS trajectory (yellow line) with a small surround and a reference box attached at the end of the trajectory. Coloured areas show the ROIs used to build the trajectory. Figure S2. Linear mixed model. a. Prediction of the fraction of SLS acquisitions to discard as a function of the number of pixels in the ROI’s surround region (gray area represents 95% confidence intervals). The model shows that scanning larger surrounds reduces the number of SLS lines to discard adding robustness to SLS acquisitions. b. QQ plot of the residuals of the data after fitting the linear mixed model to model the fraction of discarded SLS acquisitions. Since normality is a necessary condition to apply linear mixed models, we re-fitted the model after removing one outlier and observed that results were similar to the ones obtained using the full data set (beta1 = −0.11, p value=0.048). Table S1. Mean ± SEM (and p values for Wilcoxon signed rank test) for SNR and pairwise correlations of different processing pipeline reported in Fig. 6. [file 40708_2022_166_MOESM1_ESM.docx]

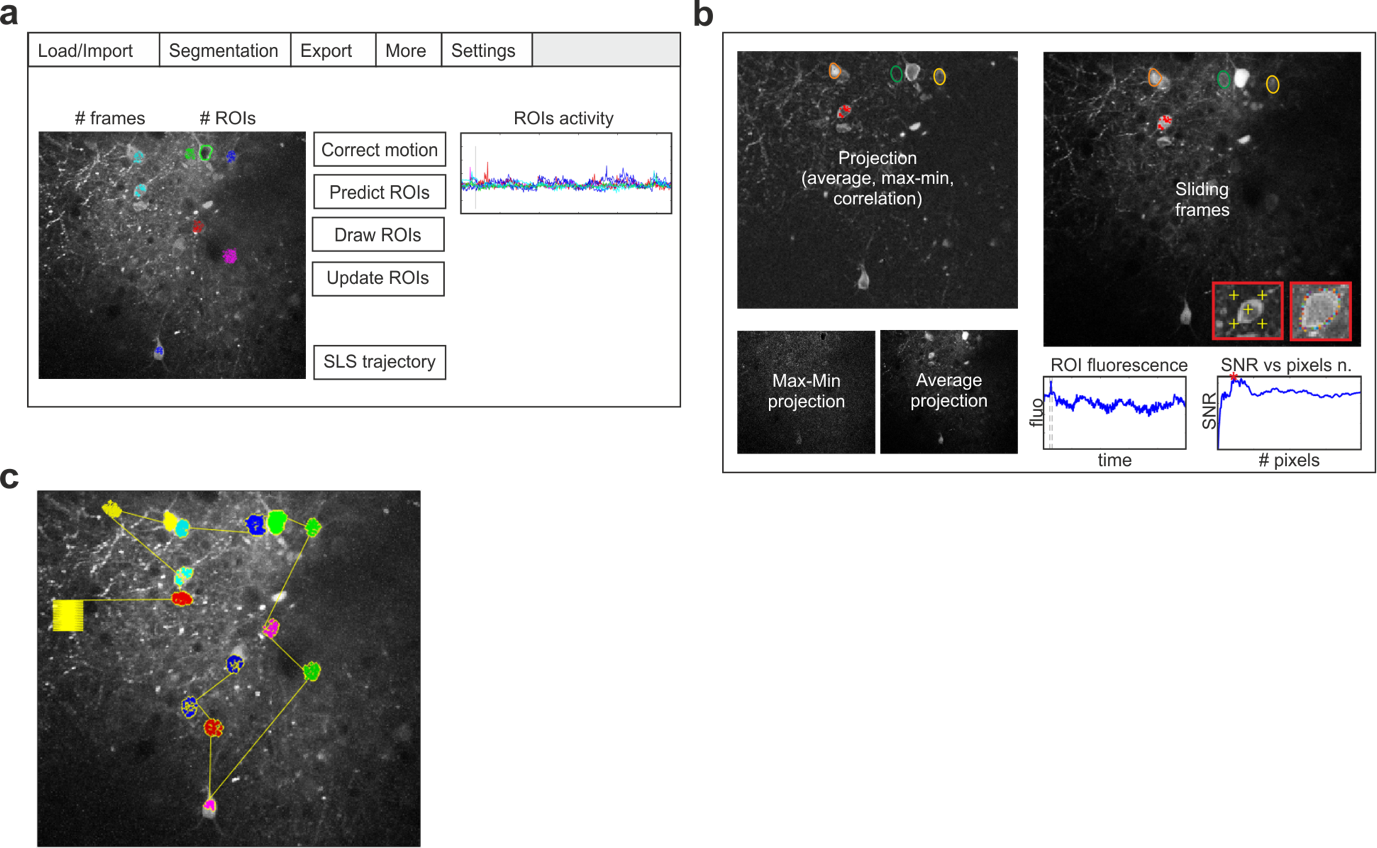


Supplementary Figure 1. **SmaRT2P graphical user interface**. **a**. Schematic of the main GUI window of SmaRT2P. In this window users can visualize the raster movie, the segmented ROIs, and the extracted activity. **b**. Schematic of the GUI window for the segmentation of raster data. Users can visualize simultaneously multiple projections of the acquisition (left) and scroll through single frames (right). Users can segment ROIs using a bounding box (red inset, left) or manually drawing the contour (red inset, right) both on the large projection window and on the sliding frames. The smart selection of the pixels includes in the segmentation only those pixels that maximize the extracted SNR (bottom right), but users can adjust the pixels selection manually. **c**. Example SLS trajectory (yellow line) with a small surround and a reference box attached at the end of the trajectory. Coloured areas show the ROIs used to build the trajectory.


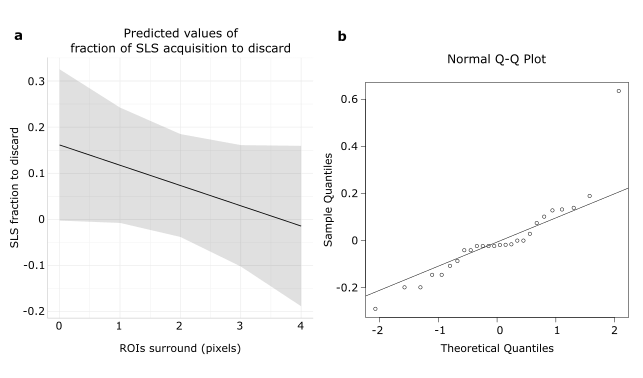


Supplementary Figure 2. **Linear mixed model**. **a**. Prediction of the fraction of SLS acquisitions to discard as a function of the number of pixels in the ROI’s surround region (gray area represents 95% confidence intervals). The model shows that scanning larger surrounds reduces the number of SLS lines to discard adding robustness to SLS acquisitions. **b**. QQ plot of the residuals of the data after fitting the linear mixed model to model the fraction of discarded SLS acquisitions. Since normality is a necessary condition to apply linear mixed models, we re-fitted the model after removing one outlier and observed that results were similar to the ones obtained using the full dataset (beta1 = -0.11, p-val=0.048).

**Supplementary Table 1**. Mean ± SEM (and p-values for Wilcoxon signed rank test) for SNR and pairwise correlations of different processing pipeline reported in Figure 6.

| Anesthetized. Surround = 0, n=101 | Δ SNR | Δ Pairwise correlations |
| --- | --- | --- |
|  | mean±SEM (p-val) | mean±SEM (p-val) |
| Local neuropil | -4.62±0.60 (6e-13) | -0.501±0.012 (3e-18) |
| PCA neuropil | -3.42±0.36 (2e-14) | -0.512±0.020 (4e-18) |
| SNR-motion | 1.21±0.05 (3e-18) | 0.012±0.001 (1e-12) |
| NoRMCorre (0.1Hz) | 0.57±1.19 (7e-6) | 0.066±0.012 (5e-4) |
| NoRMCorre (1Hz) | 4.27±1.35 (1e-1) | 0.175±0.013 (2e-17) |
| NoRMCorre | 11.39±1.04 (3e-15) | 0.211±0.015 (1e-17) |
| SNR-motion + Local neuropil | 1.67±0.07 (3e-18) | -0.018±0.002 (2e-13) |
| SNR-motion + PCA neuropil | -1.66±2.05 (4e-10) | -0.431±0.015 (3e-18) |
| NoRMCorre (0.1Hz) + Local neuropil | -0.02±2.03 (7e-6) | -0.316±0.018 (1e-17) |
| NoRMCorre (1Hz) + Local neuropil | 28.27±22.38 (2e-1) | -0.200±0.018 (1e-13) |
| NoRMCorre + Local neuropil | -2.96±0.38 (3e-11) | -0.517±0.020 (4e-18) |
| NoRMCorre (0.1Hz) + PCA neuropil | -3.57±0.34 (6e-15) | -0.524±0.018 (3e-18) |
| NoRMCorre (1Hz) + PCA neuropil | -3.80±0.35 (2e-15) | -0.541±0.015 (3e-18) |
| NoRMCorre + PCA neuropil | -6.89±0.36 (3e-18) | -0.551±0.014 (3e-18) |
| Background | -1.18±0.21 (2e-7) | -0.329±0.010 (3e-18) |
| Background + Local neuropil subtract | 17.03±15.03 (2e-11) | -0.546±0.014 (3e-18) |
| Background + PCA neuropil subtract | -3.28±0.37 (2e-13) | -0.505±0.018 (3e-18) |
| Background + SNR-motion | 0.10±0.21 (8e-1) | -0.313±0.010 (3e-18) |
| Background + NoRMCorre (0.1Hz) | -1.73±1.09 (8e-17) | -0.128±0.024 (3e-6) |
| Background + NoRMCorre (1Hz) | 2.29±1.35 (8e-1) | 0.102±0.021 (3e-6) |
| Background + NoRMCorre | 9.83±1.01 (1e-13) | 0.192±0.017 (1e-15) |
| Background + SNR-motion + Local neuropil | 0.58±0.21 (1e-2) | -0.323±0.010 (3e-18) |
| Background + SNR-motion + PCA neuropil | 15.30±14.13 (5e-11) | -0.471±0.017 (3e-18) |
| Background + NoRMCorre (0.1Hz) + Local neuropil | 55.18±49.30 (3e-5) | -0.347±0.019 (8e-18) |
| Backgr subtract + NoRMCorre (1Hz) + Local neuropil | 3.19±2.80 (1e-2) | -0.213±0.019 (6e-14) |
| Backgr subtract + NoRMCorre + Local neuropil | -2.50±0.39 (1e-8) | -0.511±0.018 (3e-18) |
| Backgr subtract + NoRMCorre (0.1Hz) + PCA neuropil | -3.75±0.36 (3e-15) | -0.529±0.018 (3e-18) |
| Backgr subtract + NoRMCorre (1Hz) + PCA neuropil | -4.15±0.37 (1e-15) | -0.556±0.015 (3e-18) |
| Backgr subtract + NoRMCorre + PCA neuropil | -7.14±0.38 (3e-18) | -0.567±0.014 (3e-18) |

| Anesthetized. Surround = 1, n=33 | Δ SNR | Δ Pairwise correlation |
| --- | --- | --- |
|  | mean±SEM (p-val) | mean±SEM (p-val) |
| Local neuropil | 15.12±20.48 (6e-5) | -0.577±0.012 (5e-7) |
| PCA neuropil | -4.05±0.60 (7e-6) | -0.624±0.022 (5e-7) |
| SNR-motion | 2.21±0.17 (5e-7) | 0.000±0.002 (8e-1) |
| NoRMCorre (0.1Hz) | -1.71±0.49 (5e-7) | 0.007±0.015 (6e-3) |
| NoRMCorre (1Hz) | -0.78±1.62 (2e-2) | 0.036±0.017 (5e-1) |
| NoRMCorre | 4.08±0.87 (7e-5) | -0.007±0.020 (3e-1) |
| SNR-motion + Local neuropil | 3.05±0.24 (5e-7) | -0.041±0.003 (5e-7) |
| SNR-motion + PCA neuropil | 0.51±5.61 (4e-5) | -0.539±0.023 (5e-7) |
| NoRMCorre (0.1Hz) + Local neuropil | 1.34±4.95 (7e-4) | -0.468±0.032 (6e-7) |
| NoRMCorre (1Hz) + Local neuropil | 54.80±42.42 (3e-1) | -0.383±0.021 (6e-7) |
| NoRMCorre + Local neuropil | -3.61±0.62 (3e-5) | -0.621±0.023 (5e-7) |
| NoRMCorre (0.1Hz) + PCA neuropil | -4.39±0.57 (2e-6) | -0.626±0.022 (5e-7) |
| NoRMCorre (1Hz) + PCA neuropil | -4.33±0.53 (2e-6) | -0.640±0.017 (5e-7) |
| NoRMCorre + PCA neuropil | -6.05±0.42 (5e-7) | -0.652±0.014 (5e-7) |
| Background | -1.94±0.51 (2e-3) | -0.420±0.012 (5e-7) |
| Background + Local neuropil subtract | -3.28±2.24 (3e-4) | -0.652±0.015 (5e-7) |
| Background + PCA neuropil subtract | -4.14±0.63 (8e-6) | -0.611±0.024 (5e-7) |
| Background + SNR-motion | 0.46±0.56 (9e-1) | -0.397±0.011 (5e-7) |
| Background + NoRMCorre (0.1Hz) | -4.05±0.57 (3e-6) | -0.355±0.034 (2e-6) |
| Background + NoRMCorre (1Hz) | -3.74±1.54 (4e-3) | -0.188±0.040 (3e-4) |
| Background + NoRMCorre | 2.88±1.15 (5e-3) | -0.050±0.025 (1e-2) |
| Background + SNR-motion + Local neuropil | 1.50±0.57 (3e-2) | -0.409±0.010 (5e-7) |
| Background + SNR-motion + PCA neuropil | -5.84±0.67 (9e-7) | -0.613±0.025 (5e-7) |
| Background + NoRMCorre (0.1Hz) + Local neuropil | -4.70±0.96 (6e-5) | -0.527±0.035 (5e-7) |
| Backgr subtract + NoRMCorre (1Hz) + Local neuropil | 39.35±27.25 (5e-1) | -0.407±0.024 (6e-7) |
| Backgr subtract + NoRMCorre + Local neuropil | -2.81±0.68 (6e-4) | -0.614±0.023 (5e-7) |
| Backgr subtract + NoRMCorre (0.1Hz) + PCA neuropil | -4.64±0.57 (1e-6) | -0.622±0.022 (5e-7) |
| Backgr subtract + NoRMCorre (1Hz) + PCA neuropil | -4.89±0.57 (1e-6) | -0.645±0.016 (5e-7) |
| Backgr subtract + NoRMCorre + PCA neuropil | -6.52±0.47 (5e-7) | -0.667±0.015 (5e-7) |

| Anesthetized. Surround ≥ 2, n=28 | Δ SNR | Δ Pairwise correlation |
| --- | --- | --- |
|  | mean±SEM (p-val) | mean±SEM (p-val) |
| Local neuropil | -6.44±0.48 (4e-6) | -0.487±0.012 (4e-6) |
| PCA neuropil | -4.84±0.81 (3e-5) | -0.667±0.019 (4e-6) |
| SNR-motion | 4.16±0.35 (4e-6) | -0.019±0.006 (2e-3) |
| NoRMCorre (0.1Hz) | -1.39±0.49 (4e-6) | -0.011±0.006 (7e-5) |
| NoRMCorre (1Hz) | -1.68±1.41 (2e-3) | 0.004±0.012 (3e-1) |
| NoRMCorre | 3.21±2.18 (3e-1) | -0.020±0.016 (2e-1) |
| SNR-motion + Local neuropil | 5.91±0.58 (4e-6) | -0.098±0.009 (4e-6) |
| SNR-motion + PCA neuropil | -6.59±0.47 (4e-6) | -0.489±0.012 (4e-6) |
| NoRMCorre (0.1Hz) + Local neuropil | -6.40±0.67 (7e-6) | -0.411±0.023 (4e-6) |
| NoRMCorre (1Hz) + Local neuropil | -4.54±1.83 (9e-5) | -0.285±0.021 (4e-6) |
| NoRMCorre + Local neuropil | -5.08±0.76 (7e-6) | -0.671±0.019 (4e-6) |
| NoRMCorre (0.1Hz) + PCA neuropil | -5.12±0.78 (1e-5) | -0.670±0.018 (4e-6) |
| NoRMCorre (1Hz) + PCA neuropil | -5.10±0.77 (1e-5) | -0.660±0.017 (4e-6) |
| NoRMCorre + PCA neuropil | -6.90±0.60 (4e-6) | -0.670±0.013 (4e-6) |
| Background | -1.37±0.56 (2e-2) | -0.441±0.011 (4e-6) |
| Background + Local neuropil subtract | -7.33±0.53 (4e-6) | -0.653±0.011 (4e-6) |
| Background + PCA neuropil subtract | -4.62±0.83 (4e-5) | -0.659±0.017 (4e-6) |
| Background + SNR-motion | 4.36±0.89 (2e-4) | -0.425±0.013 (4e-6) |
| Background + NoRMCorre (0.1Hz) | -3.04±0.70 (3e-4) | -0.429±0.023 (4e-6) |
| Background + NoRMCorre (1Hz) | -5.24±1.30 (7e-5) | -0.284±0.038 (4e-6) |
| Background + NoRMCorre | 0.46±1.97 (9e-1) | -0.120±0.030 (4e-6) |
| Background + SNR-motion + Local neuropil | 5.96±1.02 (4e-5) | -0.457±0.013 (4e-6) |
| Background + SNR-motion + PCA neuropil | -7.36±0.49 (4e-6) | -0.640±0.015 (4e-6) |
| Background + NoRMCorre (0.1Hz) + Local neuropil | -7.68±0.54 (4e-6) | -0.515±0.029 (4e-6) |
| Backgr subtract + NoRMCorre (1Hz) + Local neuropil | -4.30±2.75 (8e-4) | -0.338±0.026 (4e-6) |
| Backgr subtract + NoRMCorre + Local neuropil | -2.57±0.94 (2e-2) | -0.669±0.017 (4e-6) |
| Backgr subtract + NoRMCorre (0.1Hz) + PCA neuropil | -5.03±0.79 (1e-5) | -0.664±0.017 (4e-6) |
| Backgr subtract + NoRMCorre (1Hz) + PCA neuropil | -5.61±0.73 (6e-6) | -0.672±0.016 (4e-6) |
| Backgr subtract + NoRMCorre + PCA neuropil | -7.49±0.64 (4e-6) | -0.692±0.013 (4e-6) |

| Awake. Surround = 0, n=16 | Δ SNR | Δ Pairwise correlations |
| --- | --- | --- |
|  | mean±SEM (p-val) | mean±SEM (p-val) |
| Local neuropil | -8.87±4.96 (0.0023) | -0.368±0.039 (0.0004) |
| PCA neuropil | -7.75±5.70 (0.0004) | -0.338±0.054 (0.0005) |
| SNR-motion | 1.09±0.12 (0.0004) | 0.005±0.002 (0.0703) |
| NoRMCorre (0.1Hz) | 3.80±13.48 (0.1961) | 0.152±0.050 (0.0200) |
| NoRMCorre (1Hz) | 13.82±5.41 (0.0151) | 0.230±0.043 (0.0019) |
| NoRMCorre | 7.30±5.59 (0.4380) | 0.273±0.035 (0.0005) |
| SNR-motion + Local neuropil | 1.25±0.38 (0.0072) | -0.016±0.003 (0.0006) |
| SNR-motion + PCA neuropil | -1.82±9.37 (0.0879) | -0.288±0.050 (0.0016) |
| NoRMCorre (0.1Hz) + Local neuropil | -4.51±5.23 (0.3520) | -0.211±0.044 (0.0027) |
| NoRMCorre (1Hz) + Local neuropil | 21.96±21.93 (0.7564) | -0.153±0.039 (0.0023) |
| NoRMCorre + Local neuropil | -7.15±5.73 (0.0013) | -0.339±0.055 (0.0005) |
| NoRMCorre (0.1Hz) + PCA neuropil | -8.15±5.23 (0.0004) | -0.324±0.044 (0.0004) |
| NoRMCorre (1Hz) + PCA neuropil | -7.01±4.62 (0.0011) | -0.334±0.036 (0.0004) |
| NoRMCorre + PCA neuropil | -8.24±4.96 (0.0016) | -0.357±0.037 (0.0004) |
| Background | -5.25±4.40 (0.0023) | -0.199±0.040 (0.0019) |
| Background + Local neuropil subtract | -9.89±5.76 (0.0016) | -0.388±0.046 (0.0004) |
| Background + PCA neuropil subtract | -6.09±3.91 (0.0005) | -0.305±0.038 (0.0004) |
| Background + SNR-motion | -4.17±4.44 (0.4691) | -0.196±0.041 (0.0019) |
| Background + NoRMCorre (0.1Hz) | 2.89±14.09 (0.0113) | 0.095±0.064 (0.1961) |
| Background + NoRMCorre (1Hz) | 8.62±7.16 (0.0879) | 0.195±0.056 (0.0061) |
| Background + NoRMCorre | 2.19±7.10 (0.5014) | 0.265±0.037 (0.0006) |
| Background + SNR-motion + Local neuropil | -3.81±4.56 (0.0627) | -0.210±0.040 (0.0013) |
| Background + SNR-motion + PCA neuropil | -5.76±7.14 (0.0787) | -0.299±0.052 (0.0013) |
| Background + NoRMCorre (0.1Hz) + Local neuropil | -2.15±6.32 (0.6417) | -0.221±0.046 (0.0027) |
| Backgr subtract + NoRMCorre (1Hz) + Local neuropil | 1.98±7.54 (0.4080) | -0.161±0.041 (0.0019) |
| Backgr subtract + NoRMCorre + Local neuropil | -5.28±3.95 (0.0038) | -0.308±0.040 (0.0004) |
| Backgr subtract + NoRMCorre (0.1Hz) + PCA neuropil | -8.74±5.50 (0.0005) | -0.338±0.046 (0.0004) |
| Backgr subtract + NoRMCorre (1Hz) + PCA neuropil | -7.72±4.96 (0.0008) | -0.344±0.040 (0.0004) |
| Backgr subtract + NoRMCorre + PCA neuropil | -8.71±5.33 (0.0023) | -0.373±0.041 (0.0004) |

| Awake. Surround ≥ 1, n=12 | Δ SNR | Δ Pairwise correlations |
| --- | --- | --- |
|  | mean±SEM (p-val) | mean±SEM (p-val) |
| Local neuropil | -7.34±3.06 (0.0161) | -0.449±0.041 (0.0005) |
| PCA neuropil | -7.37±2.94 (0.0005) | -0.449±0.063 (0.0005) |
| SNR-motion | 4.25±1.34 (0.0005) | 0.005±0.012 (0.2334) |
| NoRMCorre (0.1Hz) | -1.72±3.72 (0.0771) | 0.015±0.015 (0.9097) |
| NoRMCorre (1Hz) | 5.63±3.36 (0.1514) | 0.150±0.035 (0.0010) |
| NoRMCorre | 12.19±2.73 (0.0024) | 0.134±0.029 (0.0010) |
| SNR-motion + Local neuropil | 5.14±1.55 (0.0005) | -0.029±0.016 (0.0269) |
| SNR-motion + PCA neuropil | -4.27±5.56 (0.0342) | -0.410±0.041 (0.0005) |
| NoRMCorre (0.1Hz) + Local neuropil | -1.44±4.66 (0.2036) | -0.224±0.060 (0.0093) |
| NoRMCorre (1Hz) + Local neuropil | 36.81±37.73 (0.6221) | -0.195±0.072 (0.0210) |
| NoRMCorre + Local neuropil | -5.51±3.35 (0.1099) | -0.451±0.065 (0.0005) |
| NoRMCorre (0.1Hz) + PCA neuropil | -7.66±2.68 (0.0005) | -0.458±0.061 (0.0005) |
| NoRMCorre (1Hz) + PCA neuropil | -7.18±2.51 (0.0005) | -0.471±0.054 (0.0005) |
| NoRMCorre + PCA neuropil | -10.24±3.11 (0.0005) | -0.494±0.054 (0.0005) |
| Background | -3.16±1.63 (0.0005) | -0.296±0.048 (0.0005) |
| Background + Local neuropil subtract | -8.82±3.44 (0.0049) | -0.520±0.057 (0.0005) |
| Background + PCA neuropil subtract | -6.81±2.85 (0.0005) | -0.439±0.062 (0.0005) |
| Background + SNR-motion | 1.29±2.49 (0.7334) | -0.269±0.055 (0.0034) |
| Background + NoRMCorre (0.1Hz) | -4.90±4.84 (0.0269) | -0.179±0.057 (0.0093) |
| Background + NoRMCorre (1Hz) | 1.24±4.03 (0.5186) | 0.127±0.043 (0.0161) |
| Background + NoRMCorre | 9.51±2.79 (0.0093) | 0.117±0.033 (0.0049) |
| Background + SNR-motion + Local neuropil | 2.20±2.64 (0.4697) | -0.291±0.050 (0.0015) |
| Background + SNR-motion + PCA neuropil | -9.91±3.41 (0.0010) | -0.475±0.059 (0.0005) |
| Background + NoRMCorre (0.1Hz) + Local neuropil | -1.55±5.83 (0.5186) | -0.258±0.071 (0.0093) |
| Backgr subtract + NoRMCorre (1Hz) + Local neuropil | -0.09±8.06 (0.5186) | -0.214±0.077 (0.0210) |
| Backgr subtract + NoRMCorre + Local neuropil | -4.55±3.24 (0.1514) | -0.444±0.061 (0.0005) |
| Backgr subtract + NoRMCorre (0.1Hz) + PCA neuropil | -7.73±2.65 (0.0005) | -0.456±0.062 (0.0005) |
| Backgr subtract + NoRMCorre (1Hz) + PCA neuropil | -7.92±2.81 (0.0005) | -0.499±0.060 (0.0005) |
| Backgr subtract + NoRMCorre + PCA neuropil | -10.54±3.27 (0.0005) | -0.519±0.059 (0.0005) |
